# Sex differences in the prevalence and adverse outcomes of sarcopenia and sarcopenic obesity in community dwelling elderly in East China using the AWGS criteria

**DOI:** 10.1186/s12902-019-0432-x

**Published:** 2019-10-25

**Authors:** Yanping Du, Xiaodong Wang, Hua Xie, Songbai Zheng, Xiaoqing Wu, Xiaoying Zhu, Xuemei Zhang, Sihong Xue, Huilin Li, Wei Hong, Wenjing Tang, Minmin Chen, Qun Cheng, Jianqin Sun

**Affiliations:** 1 Department of Osteoporosis and Bone Disease, Fudan University affiliated Huadong Hospital, 221 West Yan An Road, Shanghai, 200040 China; 2 Research Section of Geriatric Metabolic Bone Disease, Shanghai Geriatric Institute, Shanghai, China; 3 National Clinical Research Center for Aging and Medicine, Fudan University, Shanghai, China; 4 The Zhoujiaqiao Community Health Service Center, Shanghai, China; 5 Nutrition Department, Fudan University affiliated Huadong Hospital, Shanghai, China, 221 West Yan An Road, Shanghai, 200040 China

**Keywords:** Sarcopenia, Obesity, Sex, Prevalence, Outcome, Gender

## Abstract

**Background:**

Sarcopenia and sarcopenic obesity (SO) have a greater impact on the elderly. This study aimed to explore whether there were sex differences in the prevalence and adverse outcomes of sarcopenia and SO in community-dwelling elderly individuals in East China.

**Methods:**

This was a cross-sectional study that enrolled 213 males and 418 females aged > 65 years. Demographic characteristics, body composition, hand grip, gait speed, and indices of glucose and lipid metabolism were collected. Sarcopenia and SO were diagnosed using the Asian Working Group for Sarcopenia criteria.

**Results:**

(1) The prevalence of sarcopenia was 19.2% in males and 8.6% in females. The prevalence of SO was 7.0% in males and 2.4% in females. (2) In males, the odds ratios (ORs) of osteoporosis and dyslipidemia in the SO group were 4.21-fold and 4.15-fold higher than those in the normal group, respectively. In females, the ORs of osteoporosis and hyperglycemia in the SO group were 1.12-fold and 4.21-fold higher than those in the normal group.

**Conclusions:**

Males were more likely to be sarcopenic and to have SO than females using the AWGS criteria. Females with SO were more likely to have higher blood glucose, whereas males with SO were more likely to have osteoporosis and dyslipidemia.

## Background

Asia is the fastest aging region in the world, and China has the largest elderly population in Asia. Therefore, aging-related diseases, such as sarcopenia, will have a greater impact on Chinese elderly individuals than on those in other countries. The term sarcopenia, first described by Rosenberg in 1989, was initially used to describe aged-related loss of lean body mass [1]. Sarcopenia is considered to be associated with many adverse outcomes, such as increased risk of falls and fractures, impaired cardiopulmonary performance, disability, and mortality [2]. Furthermore, recent studies have reported specific features of sarcopenia characterized by excess body fat and reduced muscle mass and strength, which have been termed “sarcopenic obesity” (SO) [3]. Greater attention should be paid to people with SO because they are at higher risk for adverse outcomes, including metabolic diseases, cardiovascular diseases and a significant increase in mortality, than those with pure sarcopenia [4].

Our previous studies and other studies have shown that elderly males have more muscle mass than elderly females, but their muscle deterioration is faster, while elderly females have more fat mass [5–8]. Moreover, this sex difference in the change in muscle mass and fat mass has a different impact on bone mineral density (BMD) and hip geometric structure. To date, it remains unclear whether this difference in body composition with age between males and females leads to sex differences in SO, which is characterized by a combination of muscle degeneration and increased fat. The aims of this study were (1) to compare the prevalence of sarcopenia and SO in elderly males and females in a community-dwelling elderly population in East China; (2) to explore whether the changes in muscle mass and fat mass between the SO subjects and normal subjects had sex differences; and (3) to explore whether there are sex differences in adverse clinical events related to SO, including osteoporosis, hyperglycemia, and dyslipidemia.

Our study is unique in the following three aspects. First, only three studies have been published regarding SO in China [9–11]; two were performed in males, and the other was performed in females. However, both sexes were compared in our study. Second, none of the three studies used the Asian Working Group for Sarcopenia (AWGS) obesity definition. There are currently at least six diagnostic criteria developed by international groups, such as the European Working Group on Sarcopenia in Older People (EWGSOP) [12] the AWGS [13], the International Working Group on Sarcopenia [14], and the Foundation for the National Institutes of Health Sarcopenia Project [15]. It is clear that the prevalence and related adverse outcomes vary when using different diagnostic criteria. The universal perspective is that the diagnostic criteria proposed by the AWGS are more suitable for the Asian population because the cut-off values are based on published data from Japan, Korea, China, Malaysia, and other countries. Therefore, the AWGS diagnostic criteria were used in our study. Third, we performed a close examination of adverse outcomes related to SO in both sexes to determine the sex-specific influence.

## Methods

### Subjects

We recruited community-dwelling older adults from the ZHOUJIAQIAO Community, Shanghai, China between April 2016 and March 2017. The inclusion criteria were being 65–89 years of age, living in the community, and having the ability to walk independently. Subjects with the following conditions were excluded: (1) severe cognitive impairment; (2) a history of hyperlipidemia, diabetes mellitus, severe renal or hepatic disease; (3) use of medications that might affect bone metabolism, glucose metabolism or lipid metabolism; (4) implanted pacemakers; and (5) clinically visible edema that could alter the bioelectrical impedance analysis (BIA) results. This study protocol and procedures were approved by the ethics committee of the hospital (No: 2014 K004). Written informed consent was obtained from all participants included in the study.

### Collection of basic information and anthropometric measurements

All participants completed a questionnaire that included questions regarding age, sex, physical exercise (> 3 times per week for > 30 min each time), dairy intake (average daily milk consumption ≥250 ml), and medical history. Height was measured using a stadiometer to the nearest 0.01 m. Body weight was measured with a standard balance beam scale to the nearest 0.1 kg. Body mass index (BMI) was calculated as body weight divided by the square of the height (kg/m^2^).

### Muscle mass and fat mass measurements

Muscle mass was measured by BIA using the Inbody720 (Biospace Corp., Seoul, Korea). Whole-body impedance was measured using an ipsilateral foot-hand electrical pathway. Subjects were required to fast for 4 h and abstain from alcohol for 8 h prior to the test. Appendicular skeletal mass (ASM) was defined as the sum of the lean soft tissue masses of the arms and legs. The ASM was adjusted using the appendicular skeletal muscle index (ASMI = ASM/height^2^) as described by Tanimoto [16].

### Measurement of hand grip strength

Hand grip strength was measured three times with a Jamar mechanical dynamometer on the dominant hand, and the highest value was used in the analysis, as described by Schlüssel [17]. Respondents were asked to squeeze the dynamometer with each of their hands as hard as possible. Grip force was recorded in kg. The percentile distribution of handgrip strength was calculated according to sex and age categories.

### Measurement of bone mineral density and the definition of osteoporosis

The BMD of the lumbar spine (L2–4) and the left total hip were measured with a dual-energy X-ray absorptiometry densitometer (DXA, Hologic Delphi A; Hologic Inc., Methuen, MA, USA). The precision error in our laboratory was 0.86% for the lumbar spine BMD and 1.86% for total hip BMD. The densitometer was standardized with a standard phantom before each measurement. Osteoporosis was defined at the spine and the hip separately using the T-score. Osteoporosis was defined as a T-score less than − 2.5 for either site.

### Laboratory measurements

All blood samples were obtained in the morning after a 10-h overnight fast and were stored immediately at − 80 °C for subsequent assays. Serum procollagen type 1 amino-terminal propeptide, crosslaps, and 25-hydroxyvitamin D were determined enzymatically using a chemistry analyzer (Elecsys 2010; Roche, Manheim, Germany). A glucose oxidase method was used to measure plasma glucose. Glycated hemoglobin (HbA1c) levels were assessed by a point-of-care HbA1c analyzer (Siemens DCA Vantage 3000). Cholesterol, triglycerides, high-density lipoprotein-cholesterol (HDL-C), and low-density lipoprotein-cholesterol (LDL-C) were measured in fasting blood samples using a Roche Cobas 8000 analyzer with standard automated enzymatic methodology.

Hyperglycemia was defined as fasting blood glucose > 6.1 mmol/L and a HbA1c level > 6.0%.

Dyslipidemia was defined as the presence of one or more of the following abnormal lipid profiles: hypercholesterolemia, hypo-HDL-cholesterolemia, hyper-LDL-cholesterolemia, or hypertriglyceridemia, according to the 2016 Chinese guidelines for the management of dyslipidemia in adults [18]. Hypercholesterolemia was defined as a TC level ≥ 6.2 mmol/L from a fasting blood test. HDL-C levels ≤1.0 mmol/L were defined as hypo-HDL-cholesterolemia. Hyper-LDL-cholesterolemia was defined as LDL ≥ 4.1 mmol/L. Hypertriglyceridemia was defined as a TG level ≥ 2.3 mmol/L.

### Physical performance

Gait speed was used to evaluate physical functioning; subjects walked at their usual speed with a static start without deceleration along a 6-m straight line in an examination room that was more than 8 m in length. The time was measured by the same trained study investigator. Low physical performance was defined as ≤0.8 m/s according to the AWGS definition [13].

### Definitions of sarcopenia and sarcopenic obesity

Sarcopenia was defined according to the AWGS criteria, which requires the presence of both low muscle mass and low muscle function (muscle strength or physical performance). The cut-off points for diagnosis were as follows. (1) Low muscle mass defined as an ASMI less than two standard deviations below the sex-specific normal mean of a younger reference group. According to our previous study, the cutoff values were 6.66 kg/m^2^ for males and 5.24 kg/m^2^ for females [19]; (2) Low muscle strength was defined as the lower 20% of handgrip strength in the study population with cutoff values of 24.8 kg/m^2^ for males and 15.0 kg/m^2^ for females; and (3) Poor physical performance was assessed by the 6-m walk test. Subjects with gait speeds < 0.8 m/s were considered to have poor physical performance. SO was the combination of sarcopenia and obesity. Participants were classified as obese if their percentage of body fat was above the 60th percentile of the present study sample of the same sex, which corresponded to 27.2% for males and 35.9% for females.

### Statistical analysis

Statistical analyses were carried out using SPSS software (version 20.0; SPSS Inc., Chicago, IL, USA). Data are expressed as the mean ± standard deviation, median and interquartile range (25–75%), or as a percentage. Differences between 4 groups were determined by ANOVA (continuous variables) and the chi-square test (categorical variables), where appropriate. To assess the risks of osteoporosis, hyperglycemia, and dyslipidemia based on different indices of SO, odds ratios (ORs) and 95% confidence intervals (CIs) were obtained from logistic regression models after controlling for age. A *P*-value < 0.05 was considered significant. All statistical results were based on two-sided tests.

## Results

### The prevalence of sarcopenia and sarcopenic obesity in community-dwelling elderly individuals in Shanghai

**Fig. 1:**
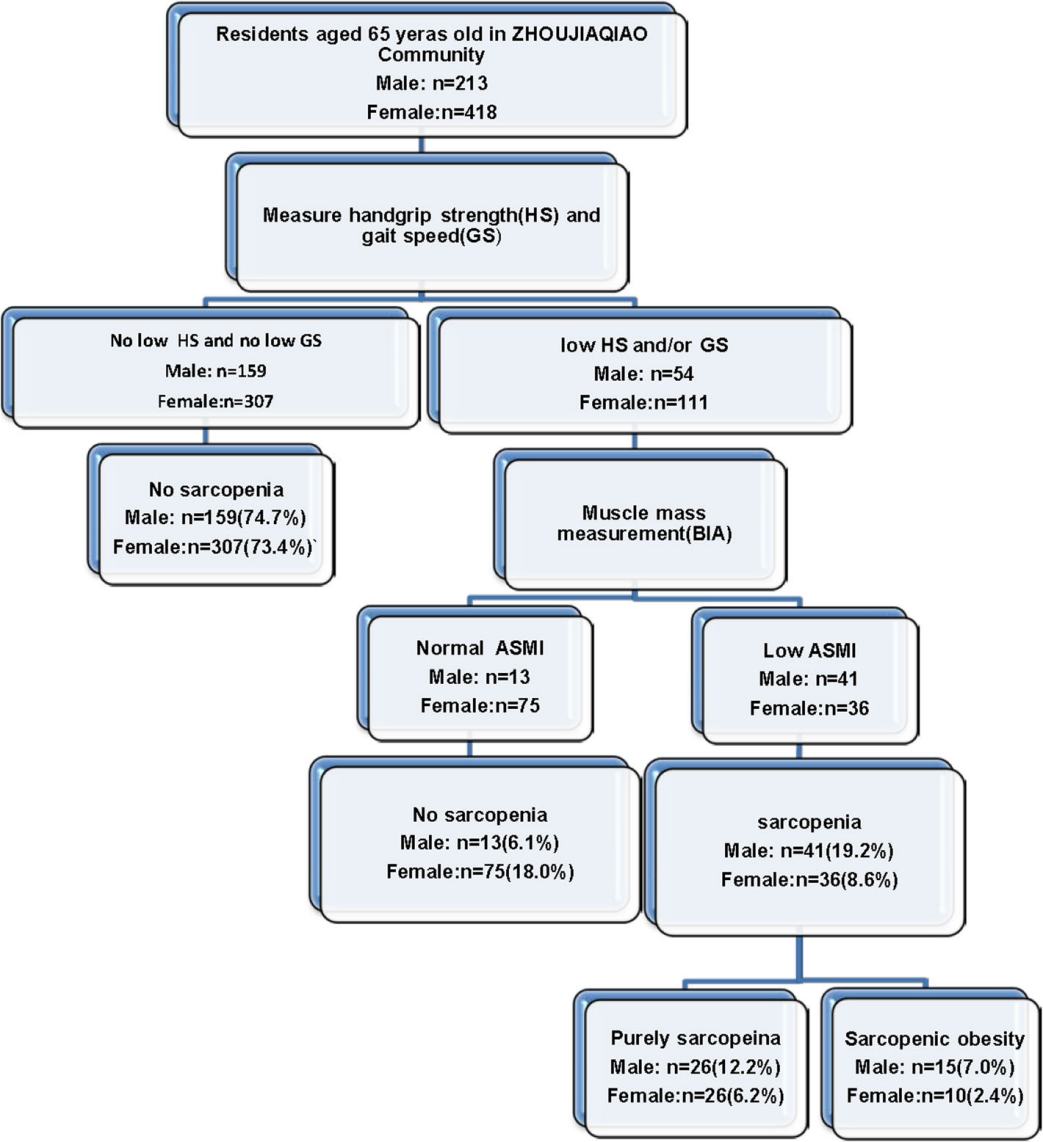
Study profile using the AWGS criteria

A total of 213 males and 418 females aged > 65 years were enrolled from the Zhoujiaqiao community in Shanghai. The prevalence of sarcopenia was 19.2% (41/213) in males and 8.6% (36/418) in females. The prevalence of SO was 7.0% (15/213) in males and 2.4% (10/418) in females, as shown in Fig. 1. Males were more likely to be sarcopenic than females (sarcopenia: OR = 2.52, 95% CI = 1.56–4.10, SO: OR = 3.10, 95% CI = 1.36–7.00).

**Fig. 2:**
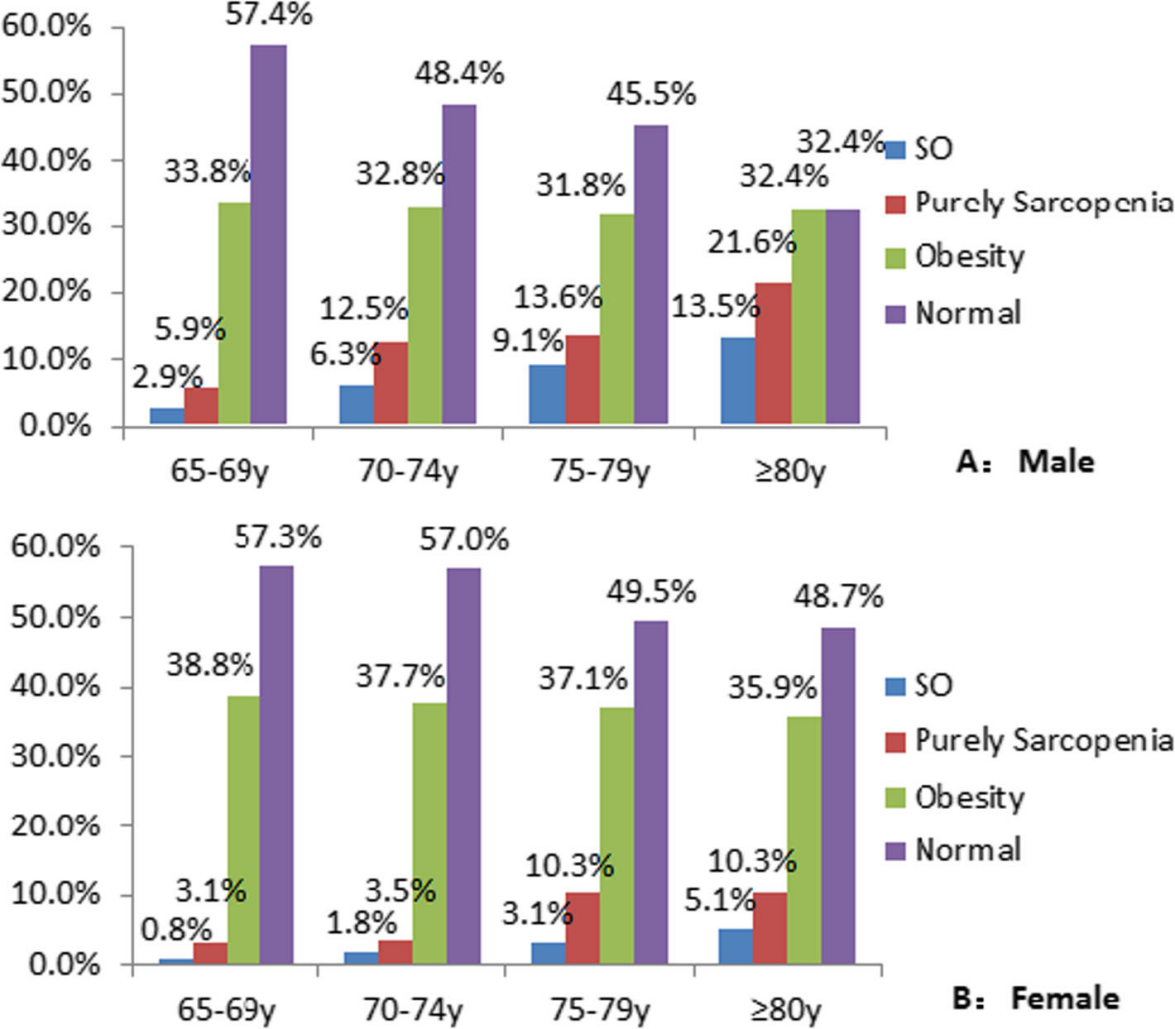
Prevalences of Sarcopenic-obesity sarcopenia, obesity by age in the community elderly in Shanghai (**a**: males **b**: females)

The prevalence of sarcopenia increased with age in both sexes (Fig. 2). The prevalence rates of pure sarcopenia and SO in males aged > 80 years were 35.1 and 13.5% and in females were 15.4 and 5.1%, respectively. In addition, the prevalence of sarcopenia in males aged 70–74 years increased significantly compared with that in males aged 65–69 years. Subsequently, the prevalence continued to increase with age, but the increasing trend became flat. The prevalence of sarcopenia clearly increased in females aged 75–79 years. Moreover, the prevalence of pure sarcopenia and SO in females was lower than that in males in all age groups. In addition, the proportion of obese people (obesity and SO) in all age groups > 65 years was mostly stable.

### Characteristics of the subjects by sarcopenia and obesity status

We classified the subjects into four sarcopenia/obesity groups according to the levels of muscle mass/muscle function and total body fat percentage. The four groups included SO, obesity (with normal muscle), sarcopenia (with normal body fat), and normal body fat and muscle, as shown in Table 1.

**Table 1:** Anthropometric and biochemical parameters among groups with SO, obesity, or sarcopenia in both sexes

	Sex	SO	Obesity	Sarcopenia	Normal	*P*
Age (years)	M	75.3 ± 6.1^a^	73.3 ± 7.4 ^a^	73.8 ± 8.2 ^a^	71.6 ± 9.1 ^a^	0.343
	F	71.4 ± 5.4 ^a^	67.2 ± 7.3 ^a^	72.4 ± 7.7 ^a^	68.1 ± 8.1 ^a^	0.175
Physical exercise (%)	M	33.3 ^a^	37.1 ^a^	38.5 ^a^	43.1 ^a^	0.811
	F	30.0 ^b^	25.4 ^b^	30.8 ^b^	49.3 ^a^	0.000
Dairy intake (%)	M	26.6 ^a^	27.1 ^a^	30.8 ^a^	28.4 ^a^	0.986
	F	30.0 ^a^	28.0 ^a^	34.6 ^a^	32.9 ^a^	0.741
BMI (kg/m^2^)	M	23.5 ± 2.0 ^b^	26.4 ± 2.4 ^a^	21.0 ± 2.3 ^b^	23.0 ± 2.6 ^b^	0.000
	F	23.9 ± 1.7 ^b^	26.3 ± 3.3 ^a^	18.8 ± 1.5 ^d^	21.8 ± 2.1 ^bc^	0.000
Total body fat percentage (%)	M	31.3 ± 2.7 ^a^	31.1 ± 2.9 ^a^	21.7 ± 6.7 ^b^	22.4 ± 4.3 ^b^	0.000
	F	43.2 ± 5.0 ^a^	39.8 ± 3.6 ^a^	28.4 ± 4.9 ^b^	29.8 ± 4.7 ^b^	0.000
ASMI (kg/m^2^)	M	5.84 ± 0.56 ^b^	7.74 ± 0.59 ^a^	6.39 ± 0.53 ^b^	7.69 ± 0.65 ^a^	0.000
	F	4.48 ± 0.98 ^b^	6.41 ± 0.68 ^a^	4.96 ± 0.23 ^b^	6.19 ± 0.63 ^a^	0.000
Hand grip (kg)	M	24.2 ± 4.7 ^b^	36.9 ± 7.7 ^a^	26.7 ± 5.9 ^b^	33.8 ± 6.7 ^a^	0.002
	F	18.6 ± 5.5 ^ac^	19.0 ± 4.8 ^ab^	16.3 ± 5.5 ^bc^	20.3 ± 5.0 ^a^	0.001
Spine BMD (g/cm^2^)	M	0.92 ± 0.15 ^b^	1.07 ± 0.19 ^a^	1.00 ± 0.17 ^a^	0.99 ± 0.16 ^a^	0.035
	F	0.82 ± 0.12 ^b^	1.04 ± 0.16 ^a^	0.68 ± 0.16 ^c^	0.83 ± 0.16 ^b^	0.001
Hip BMD (g/cm^2^)	M	0.81 ± 0.13 ^b^	1.03 ± 0.12 ^a^	0.77 ± 0.12 ^b^	0.89 ± 0.10 ^b^	0.003
	F	0.73 ± 0.11 ^b^	0.89 ± 0.13 ^a^	0.61 ± 0.15 ^c^	0.76 ± 0.13 ^b^	0.015
PINP	M	29.8 ± 9.9 ^a^	36.9 ± 10.4 ^a^	38.6 ± 15.6 ^a^	34.5 ± 12.4 ^a^	0.884
	F	46.4 ± 11.1 ^a^	51.7 ± 12.3 ^a^	51.7 ± 19.2 ^a^	50.2 ± 17.7 ^a^	0.975
β-CTX	M	293 ± 116 ^a^	316 ± 121 ^a^	355 ± 161 ^a^	273 ± 148 ^a^	0.403
	F	449 ± 117 ^a^	415 ± 137 ^a^	495 ± 145 ^a^	448 ± 195 ^a^	0.525
25OHD	M	18.6 ± 5.8 ^a^	21.0 ± 7.6 ^a^	19.6 ± 4.3 ^a^	22.0 ± 7.7 ^a^	0.484
	F	21.0 ± 5.5 ^a^	18.3 ± 6.4 ^a^	22.4 ± 5.1 ^a^	22.1 ± 7.0 ^a^	0.161
Fasting glucose (mmol/L)	M	5.33 ± 0.57 ^a^	5.65 ± 1.12 ^a^	5.12 ± 0.52 ^a^	5.30 ± 0.98 ^a^	0.656
	F	6.14 ± 0.53 ^a^	5.88 ± 0.63 ^ab^	4.86 ± 0.45 ^c^	5.23 ± 0.64 ^bc^	0.001
HbA1C (%)	M	5.30 ± 0.59 ^a^	5.80 ± 0.44 ^a^	5.09 ± 0.69 ^a^	5.16 ± 0.49 ^a^	0.324
	F	6.23 ± 0.66 ^a^	6.01 ± 0.61 ^a^	5.11 ± 0.71 ^b^	4.91 ± 0.48 ^b^	0.011
Total cholesterol (mmol/L)	M	6.4 (5.41–7.12) ^a^	5.90 (4.95–6.99) ^ab^	5.55 (4.35–6.22) ^bc^	5.33 (4.52–5.61) ^c^	0.012
	F	6.11 (5.82–6.78) ^a^	5.65 (5.11–6.52) ^a^	5.17 (4.88–6.19) ^b^	5.78 (5.21–6.51) ^a^	0.023
Triglycerides (mmol/L)	M	2.40 (1.3–2.6) ^a^	2.47 (1.98–2.88) ^a^	1.60 (1.13–2.21) ^b^	1.63 (1.21–2.38) ^b^	0.021
	F	1.68 (1.12–2.11) ^a^	1.88 (1.49–2.62) ^a^	1.78 (1.41–2.58) ^a^	1.63 (1.18–2.18) ^a^	0.523
HDL cholesterol (mmol/L)	M	1.50 (1.28–1.70) ^ab^	1.09 (0.81–1.36) ^b^	1.75 (1.49–1.88) ^a^	1.26 (0.99–1.48) ^b^	0.045
	F	1.86 (1.58–2.49) ^a^	1.70 (1.32–2.28) ^a^	1.79 (1.33–2.25) ^a^	1.86 (1.55–2.41) ^a^	0.899
LDL cholesterol (mmol/L)	M	4.12 (3.23–4.71) ^a^	3.64 (2.60–3.91)^ab^	2.99 (2.38–3.37) ^ab^	2.82 (2.48–3.40) ^b^	0.011
	F	3.61 (3.03–4.21) ^a^	3.13 (2.89–3.81) ^a^	3.02 (2.54–3.88) ^a^	3.11 (2.69–3.72) ^a^	0.723
Speed (m/s)	M	0.82 ± 0.31 ^b^	1.03 ± 0.21 ^ab^	0.90 ± 0.29 ^b^	1.12 ± 0.22 ^a^	0.006
	F	0.79 ± 0.22 ^b^	0.89 ± 0.29 ^ab^	0.82 ± 0.21 ^b^	1.09 ± 0.27 ^a^	0.044

Notes: Values in a row with different superscript letters are significantly different, *p* < 0.05. From a to d, the mean value or the median value decreased

Abbreviations: *BMI* body mass index, *ASMI* appendicular Skeletal Mass Index, *BMD* bone mineral density, *PINP* procollagen type 1 amino-terminal propeptide, *CTX* crossLaps

The purely obese group had the highest BMD in the lumbar spine and hip among the four groups. The BMD of the male SO group was lower than that of the obesity and normal groups. The BMD of the elderly female SO group was similar to that of the normal group, which was higher than that of the purely sarcopenic group.

The SO group of females had the highest fasting blood glucose and HbA1c levels among the four groups, whereas fasting blood glucose in the male SO group was lower than that in the purely obese group but higher than that in the pure sarcopenia and normal groups.

Serum cholesterol and LDL cholesterol levels were higher in the SO group than in the other three groups in both sexes. Among females, the normal group performed more physical exercise than the other 3 groups. No significant difference was observed in bone turnover markers between the SO group and the other three groups.

### The rate of change in ASM and fat mass (FM) between the SO and normal groups in both sexes

The mean ASM in the SO group decreased by 34.7% in males and by 24.8% in females compared with that in the normal group, while FM in the SO group increased 9.6% in males and 43.8% in females, as shown in Table 2. Therefore, the rate of change in ASM/FM between the SO group and the normal group decreased 40.0% in males and 48.4% in females.

**Table 2:** Rate of change in ASM and FM between the normal group and the SO group in both sexes

		Normal group	SO group	Rate of change
Male	ASM (kg)	21.3 ± 2.7	13.9 ± 2.4	−34.7%
	FM (kg)	14.6 ± 3.6	16.0 ± 1.9	+ 9.6%
	ASM/FM	1.45	0.87	−40.0%
Female	ASM (kg)	15.1 ± 1.9	11.3 ± 3.0	−24.8%
	FM (kg)	16.2 ± 3.2	23.3 ± 2.8	+ 43.8%
	ASM/FM	0.93	0.48	−48.4%

*ASM* appendicular Skeletal Mass, *FM* fat mass

### Comparison of the odds ratios of osteoporosis, hyperglycemia, and dyslipidemia between the SO group and the other groups

**Fig. 3:**
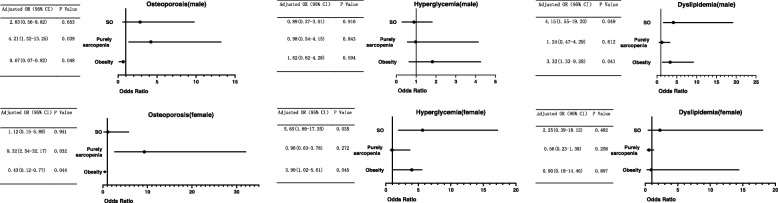
Odds ratio for predicting osteoporosis, hyperglycemia, and dyslipidemia based on SO group, sarcopenia group, and obesity group adjusted for age

The ORs of osteoporosis, hyperglycemia, and dyslipidemia in the SO group and the other groups are shown in Table 3 and Fig. 3. Obese subjects were less likely to develop osteoporosis than the subjects in the normal group. However, the OR for osteoporosis was elevated in the pure sarcopenia group. In males, the OR of osteoporosis in the SO group was 2.83-fold higher than that in the normal group. In females, the OR of osteoporosis in the SO group was slightly higher than that in the normal group.

**Table 3:** Odds ratio from logistic regression models predicting osteoporosis, hyperglycemia, and dyslipidemia based on SO group, sarcopenia group, and obesity group adjusted for age

	Characteristics	B	SE	Wald’sχ^2^	df	Odds ratio	*P* value
Male							
Osteoporosis	SO	1.04	1.12	0.86	1	2.83 (0.56–9.82)	0.653
	Purely sarcopenia	1.43	0.65	4.84	1	4.21 (1.32–13.25)	0.039
	Obesity	−0.40	0.21	3.64	1	0.67 (0.07–0.82)	0.048
	Normal					1.00(reference)	
Hyperglycemia	SO	−0.12	1.12	0.01	1	0.89 (0.27–3.01)	0.916
	Purely sarcopenia	−0.02	0.26	0.01	1	0.98 (0.54–4.15)	0.843
	Obesity	0.60	0.79	0.58	1	1.82 (0.62–4.28)	0.594
	Normal					1.00(reference)	
Dyslipidemia	SO	1.42	0.73	3.78	1	4.15 (1.55–19.20)	0.049
	Purely sarcopenia	0.22	0.42	0.27	1	1.24 (0.47–4.29)	0.612
	Obesity	1.20	0.58	4.28	1	3.32 (1.33–9.28)	0.041
	Normal					1.00(reference)	
Female							
Osteoporosis	SO	0.11	1.21	0.01	1	1.12 (0.15–5.89)	0.941
	Purely sarcopenia	2.23	0.93	5.76	1	9.32 (2.54–32.17)	0.032
	Obesity	−0.84	0.43	3.85	1	0.43 (0.12–0.77)	0.044
	Normal					1.00(reference)	
Hyperglycemia	SO	1.73	0.76	5.19	1	5.65 (1.89–17.25)	0.035
	Purely sarcopenia	−0.04	0.19	0.04	1	0.96 (0.63–3.78)	0.872
	Obesity	1.38	0.71	3.79	1	3.99 (1.02–5.61)	0.045
	Normal					1.00(reference)	
Dyslipidemia	SO	0.81	1.25	0.42	1	2.25 (0.39–18.12)	0.482
	Purely sarcopenia	−0.58	0.46	1.59	1	0.56 (0.23–1.38)	0.208
	Obesity	−0.11	0.82	0.02	1	0.90 (0.18–14.46)	0.897
	Normal					1.00(reference)	

Abbreviations: *SO* sarcopenic obesity

In females, the OR of hyperglycemia in the purely obese group and the SO group were 3.99-fold (OR = 3.99, 95% CI = 1.02–5.61) and 5.65-fold (OR = 5.65, 95% CI = 1.89–17.25) higher than that in the normal group. The OR of hyperglycemia in the SO and purely obese groups of males was similar to that in the normal group.

In addition, the OR of dyslipidemia in the pure sarcopenia group of males was similar to that in the normal group. However, in elderly males, the ORs in the purely obese group and the SO group were 3.32-fold (OR = 3.32, 95% CI = 1.33–9.28) and 4.15-fold (OR = 4.15, 95% CI = 1.55–19.20) as high as that in the normal group. No increase was observed in the ORs of the purely obese and SO groups of females.

## Discussion

This cross-sectional study was performed to explore sex differences in the prevalence of sarcopenia and SO and in the clinical adverse outcomes related to SO. Our findings demonstrate that the prevalence rates of sarcopenia and SO were 19.2 and 7.0% in elderly males and 8.6 and 2.4% in elderly females, respectively, in the Shanghai community using the AWGS criteria. The currently available literature is unclear regarding sex differences in the prevalence of sarcopenia and SO in China. Kim reported that subjects diagnosed with sarcopenia and SO among a sample of 526 community-dwelling individuals aged > 60 years were more likely to be male [20]. Hashemi and Wu also reported that male sex was independently associated with sarcopenia in community-dwelling older individuals in Iran and Taiwan [21, 22]. Other studies have reported a higher prevalence of SO in elderly males than in females [23]. The potential mechanisms leading to the sex difference in the prevalence of sarcopenia and SO include (1) males tend to lose muscle mass gradually with age, but the decline in muscle mass with age is insignificant or only slightly significant in females [24]. Muscle mass and function clearly decrease during the early stages of menopause because of the significant decrease in estrogen [25]. However, with further aging, testosterone and insulin-like growth factor-1 levels in males decrease significantly, resulting in rapid loss of muscle mass and strength, which greatly increase the risk of sarcopenia in elderly males [26]; (2) although the AWGS recommended using height-adjusted skeletal muscle mass instead of weight-adjusted skeletal muscle mass to diagnose sarcopenia, several studies have suggested that low muscle mass identified by a weight-adjusted muscle index was more suitable for overweight and obese people [27]. Therefore, the height-adjusted definition would underestimate the prevalence in females, who lose height much more during aging and have higher adiposity than males. In fact, Soo reported that the prevalence of SO in males was higher than that in females with sarcopenia defined by the ASM/height^2^, while the prevalence of SO in males was lower than that in females with sarcopenia defined by the ASM/weight [28]. However, Gao and Volpato reported a higher risk of sarcopenia in females among community-dwelling elderly adults [29, 30]. These inconsistent results may be due to differences in racial characteristics, cultural backgrounds, dietary patterns, and physical activity among the different studies.

In our study, participants were classified as obese if their percentage of body fat was above the 60th percentile among the study sample for the same sex. Therefore, 40% of subjects in both sex groups were considered obese in our study. Figure 2 shows that the proportion of obese people (obesity and SO) in all age groups > 65 years was basically stable, and there was no significant fluctuation. One of our early studies that included 2234 participants aged 21–96 years, reported that fat mass increases significantly during the perimenopausal period (41–50 years old) in females. Subsequently, the fat mass continues to increase, but the trend is flat. Fat is the main organ responsible for estrogen production and maintains estrogen at a certain level after menopause. Therefore, as a compensatory mechanism, fat mass increases significantly during perimenopause and is maintained at a high level thereafter. As a result, the cutoff point for obesity among females was higher than that among males (35.9% vs. 27.2%). In addition, the proportion of subjects with normal body composition gradually decreased with age, while the proportion of subjects with sarcopenia and SO gradually increased, suggesting that muscle degeneration was the main body composition change that accompanied aging. However, muscle degeneration was faster in males than in females. One quantitative review showed that in people older than 75 years, muscle mass decreased at a rate of 0.8–0.98% per year in males and 0.64–0.70% per year in females [31]. In addition, muscle strength was lost faster in males than in females, which decreased at a rate of 3–4% per year in males and 2.5–3% per year in females [31]. The causes that lead to the reduction in lean mass in males more rapidly are unclear. However, insulin-like growth factor 1 (IGF-1) may play an important role. IGF-1 is one of the common factors regulating muscle growth and repair. The IGF-1 level does not change with aging in females aged 65 years. Nevertheless, IGF-1 showed age-related decrease in elderly males, especially in those aged 85 years [32]. Another possible cause may be the limitation of measurements. DXA and BIA may not identify the intramuscular lipid infiltration and overestimated the muscle mass in obese people. Therefore, in elderly females with higher adiposity, DXA or BIA may overestimated their muscle mass.

Our results also show that elderly subjects with different body composition characteristics had different risks of osteoporosis, hyperglycemia, and dyslipidemia. According to previous studies, the obese population has a higher BMD and a lower risk of bone fracture; thus, they are not prone to developing osteoporosis [33]. Therefore, obesity has always been considered a protective factor for the skeleton. Our data suggest that the highest BMD was found in the lumbar vertebrae and hip of the purely obese group, and the lowest risk for osteoporosis was thereby indicated. BMD was significantly lower in the obese individuals with sarcopenia than in the purely obese group, indicating that muscle (but not fat) is the main protective factor for the skeleton. This conclusion has been verified by our preliminary study and previous studies [5, 6, 34]. Moreover, the blood glucose level increased significantly in the obese female population; the risk for hyperglycemia was higher in the SO group than in the purely obese group (ORs: 5.65 vs. 3.99). Srikanthan reported that muscle mass, independent of obesity, is significantly correlated with glucose metabolic disorders; a low muscle mass is a risk factor for early diabetes [35]. Muscle mass, muscle strength, and muscle function are all affected in diabetes, and the decrease in muscle mass and strength is positively correlated with the course of diabetes and HbA1c [36, 37]. In addition, our study also revealed that the risk of dyslipidemia in the SO group was not only higher than that in the sarcopenia group (OR: 4.15 vs. 1.24) but also higher than that in the purely obese group (OR: 4.15 vs. 3.32). Obesity is an important cause of dyslipidemia. However, the combination of obesity and muscle loss results in a higher risk of dyslipidemia. Several studies have reported a close correlation between SO and dyslipidemia. One study from Korea showed that the ORs of dyslipidemia increased by 1.12 and 1.50 in the sarcopenia and SO groups, respectively, compared with the values of a nonsarcopenia group [38]. Another study that included 1231 men aged > 70 years reported that serum triglyceride levels were higher in patients with SO than in those with pure sarcopenia and nonsarcopenic nonobesity [39]. The underlying mechanism may be related to insulin resistance [40] and the release of inflammatory cytokines [41].

Notably, this study showed that the clinical outcomes of SO varied with sex. The following results in SO females were observed compared with those in SO males: a significantly higher risk for hyperglycemia (OR: 5.65 vs. 0.89) and lower risks for osteoporosis (OR: 1.12 vs. 2.83) and dyslipidemia (OR: 2.25 vs. 4.15). These results may be related to the different body composition changes in males and females. Our results in Table 2 show that males with SO had a 34.7% decrease in appendicular skeletal muscular mass and a 9.6% increase in FM compared with the same parameters of the participants in the normal group, indicating a larger change in muscle mass than in FM. Females with SO had a 24.8% decrease in appendicular skeletal muscular mass but a 43.8% increase in FM, indicating a larger change in FM than in muscle mass. These data suggest that the occurrence of SO in males may be mainly related to a decline in muscle mass and that it may be mainly related to a fat increase in females. Both muscle mass and strength of males were greater than those of females, but the decrease in muscle mass and muscle strength of elderly males was significantly quicker than that of elderly females [31]. According to our studies and other studies, males suffer higher morbidity rates from SO than females [7, 8, 23]. Females have more FM and abdominal fat mass than males. Because of the cross-sectional nature of this study, we can only speculate from the available data that females with SO may have been initially obese and not sarcopenic. A cohort study performed by Baumgartner showed that the age-related increase in FM generally precedes the loss of muscle [42]. Another study in postmenopausal females showed that a higher BMI is a risk factor for loss of muscle mass [43]. In the present study, the cause of SO was different between males and females, leading to sex differences in adverse outcomes related to SO. Adverse outcomes were not only associated with muscle loss but also with an increase in fat. In our previous studies, we showed that FM had a positive effect on the skeleton in elderly subjects but that the effect was weaker than that of muscle mass [5, 6]. Therefore, the risk of osteoporosis in females with SO with more fat was lower than that in males. In addition, current studies have reported that either excess fat or reduced muscle can increase insulin resistance, thereby increasing the risk of diabetes and dyslipidemia. However, few studies have directly compared the effects of fat and muscle on diabetes mellitus or dyslipidemia. The present study found that females with SO with more FM were more likely to have higher blood glucose. Kawanabes reported that ASM/FM is positively correlated with the insulin sensitivity index. Our results show that the ASM/FM ratio of males with SO was higher than that of females (0.87 vs. 0.48), suggesting that females with SO have worse insulin sensitivity and therefore are more likely to have a higher blood glucose level. Moreover, males with SO with significant muscle deterioration were more likely to have dyslipidemia. Korean scholars also reported that SO is associated with an increased risk for dyslipidemia compared with the risk associated with sarcopenia or obesity alone in males. These results indicate that other factors besides insulin resistance affect dyslipidemia in elderly subjects with SO, such as sex hormones. Further studies are needed.

This study had some limitations. First, this study was cross-sectional; thus, it has a limited ability to show causal relationships. Second, all elderly subjects enrolled in this study were relatively healthy and had the ability to complete the various tests in this study; some subjects who could not complete the activities were not enrolled, which may have introduced bias in the data. Third, the subjects were not confirmed to be diabetic by an OGTT. Only a combination of an increase in blood glucose based on a single detection in fasting blood and HbA1C level was evaluated; thus, the diabetes risk data in the SO population were not obtained directly. Finally, although BIA is a commonly used and feasible tool that is recommended by the AWGS and EWGSOP for community sarcopenia assessment, the use of BIA is not the best system to investigate the body composition in the elderly population that tends to be generally dehydrated [44]. Therefore, the muscle mass measured by BIA may be underestimated in the elderly because of inadequate hydration.

## Conclusion

In conclusion, we first examined the sex differences in the prevalence and adverse outcomes of sarcopenia and SO in community-dwelling elderly subjects living in East China. Three main findings emerged. First, the prevalence rates of sarcopenia and SO were 19.2 and 7.0% in elderly males and 8.6 and 2.4% in elderly females, respectively, using the AWGS criteria. Muscle degeneration, compared with an increase of FM, was the main body compositional change in subjects > 65 years. Males were more likely to be sarcopenic or SO than females based on the AWGS criteria. Second, males in the SO group had a greater change in the ASM/FM ratio than females in the normal group, indicating that the cause of SO is different between males and females. Our results suggest that the occurrence of SO may be mainly related to muscle decline in males and a fat increase in females. Third, sex differences were observed in the clinical adverse outcomes of SO, such as osteoporosis, hyperglycemia, and dyslipidemia. Females with SO were more likely to have higher blood glucose, whereas SO males were more likely to have osteoporosis and dyslipidemia.

Therefore, different attention should be paid to the different adverse outcomes related to SO in patients of different sexes.

## Data Availability

The datasets used and/or analysed during the current study are available from the corresponding author on reasonable request.

## Abbreviations

ASM: appendicular skeletal mass
ASMI: appendicular skeletal mass index
AWGS: Asian Working Group for Sarcopenia
BIA: bioelectrical impedance analysis
BMD: bone mineral density
BMI: body mass index
CI: confidence interval
CTX: crossLaps
DXA: dual-energy X-ray absorptiometry densitomete
EWGSOP: European Working Group on Sarcopenia in Older People
FM: fat mass
GS: gait speed
HbA1c: glycated hemoglobin
HDL-C: high-density lipoprotein-cholesterol
HS: handgrip strength
IGF-1: insulin-like growth factor 1
LDL-C: low-density lipoprotein-cholesterol
OGTT: oral glucose tolerance test
OR: odds ratio
PINP: procollagen type 1 amino-terminal propeptide
SO: Sarcopenia obesity

## References

[1] Rosenberg I (1989). Summary comments: epidemiological and methodological problems in determining nutritional status of older persons. Am J Clin Nutr.

[2] Arango-Lopera VE, Arroyo P, Gutiérrez-Robledo LM, Pérez-Zepeda MU, Cesari M (2013). Mortality as an adverse outcome of sarcopenia. J Nutr Health Aging.

[3] Stenholm S, Harris TB, Rantanen T, Visser M, Kritchevsky SB, Ferrucci L (2008). Sarcopenic obesity: definition, cause and consequences. Curr Opin Clin Nutr Metab Care.

[4] Kohara K (2014). Sarcopenic obesity in aging population: current status and future directions for research. Endocrine.

[5] Du Y, Zhu H, Zheng S (2018). Age and sex effects on the relationship between body composition and hip geometric structure in males and females from East China. Archives of Osteoporosis.

[6] Cheng Q, Zhu YX, Zhang MX (2012). Age and sex effects on the association between body composition and bone mineral density in healthy Chinese men and women. Menopause..

[7] Cheng Q, Zhu X, Zhang X (2014). A cross-sectional study of loss of muscle corresponding to sarcopenia in healthy Chinese men and women: reference values, prevalence, and association with bone mass. J Bone Miner Metab.

[8] Kohara K, Ochi M, Tabara Y, Nagai T, Igase M, Miki T (2012). Arterial stiffness in sarcopenic visceral obesity in the elderly: J-SHIPP study. Int J Cardiol.

[9] Liao CD, Tsauo JY, Lin LF (2017). Effects of elastic resistance exercise on body composition and physical capacity in older women with sarcopenic obesity: A CONSORT-compliant prospective randomized controlled trial. Medicine (Baltimore).

[10] Yang CW, Li CI, Li TC (2015). Association of Sarcopenic Obesity with Higher Serum High-Sensitivity C-Reactive Protein Levels in Chinese Older Males-A Community-Based Study (Taichung Community Health Study-Elderly, TCHS-E). PLoS One.

[11] Meng P, Hu YX, Fan L (2014). Sarcopenia and sarcopenic obesity among men aged 80 years and older in Beijing: prevalence and its association with functional performance. Geriatr Gerontol Int.

[12] Cruz-Jentoft AJ, Baeyens JP, Bauer JM (2010). Sarcopenia: European consensus on definition and diagnosis: report of the European working group on sarcopenia in older people. Age Ageing.

[13] Chen LK, Liu LK, Woo J (2014). Sarcopenia in Asia: consensus report of the Asian working group for sarcopenia. J Am Med Dir Assoc.

[14] Fielding RA, Vellas B, Evans WJ (2011). Sarcopenia: An undiagnosed condition in older adults. Current consensus definition: Prevalence, etiology, and consequences. International Working Group on Sarcopenia. J Am Med Dir Assoc.

[15] Studenski SA, Peters KW, Alley DE (2014). The FNIH sarcopenia project: rationale, study description, conference recommendations, and final estimates. J Gerontol A Biol Sci Med Sci.

[16] Tanimoto Y, Watanabe M, Sun W (2012). Association between sarcopenia and higher-level functional capacity in daily living in community-dwelling elderly subjects in Japan. Arch Gerontol Geriatr.

[17] Schlüssel MM, dos Anjos LA, de Vasconcellos MT, Kac G (2008). Reference values of handgrip dynamometry of healthy adults: a population-based study. Clin Nutr.

[18] Joint committee for guideline revision (2016). Chinese guideline for the management of dyslipidemia in adults. J Geriatr Cardiol.

[19] Chen M, Bai H, Wang C (2015). Establishment of muscle mass diagnostic standard of sarcopenia using a bioelectrical impedance analysis and epidemiological investigation of the elderly in Shanghai. Chin J Geriatr.

[20] Kim TN, Yang SJ, Yoo HJ (2009). Prevalence of sarcopenia and sarcopenic obesity in Korean adults: the Korean sarcopenic obesity study. Int J Obes (Lond).

[21] Hashemi R, Shafiee G, Motlagh AD (2016). Sarcopenia and its associated factors in Iranian older individuals: results of SARIR study. Arch Gerontol Geriatr.

[22] Wu IC, Lin CC, Hsiung CA (2014). Epidemiology of sarcopenia among community-dwelling older adults in Taiwan: a pooled analysis for a broader adoption of sarcopenia assessments. Geriatr Gerontol Int.

[23] Bouchard DR, Dionne IJ, Brochu M (2009). Sarcopenic/obesity and physical capacity in older men and women: data from the nutrition as a determinant of successful aging (NuAge)-the Quebec longitudinal study. Obesity (Silver Spring).

[24] Shimokata H, Ando F, Yuki A, Otsuka R (2014). Age-related changes in skeletal muscle mass among community-dwelling Japanese: a 12-year longitudinal study. Geriatr Gerontol Int.

[25] Tiidus PM (2011). Benefits of estrogen replacement for skeletal muscle mass and function in post-menopausal females: evidence from human and animal studies. Eurasian J Med.

[26] Yamada M, Nishiguchi S, Fukutani N (2013). Prevalence of sarcopenia in community-dwelling Japanese older adults. J Am Med Dir Assoc.

[27] Lee WJ, Liu LK, Peng LN, Lin MH, Chen LK (2013). ILAS Research Group. Comparisons of sarcopenia defined by IWGS and EWGSOP criteria among older people: results from the I-Lan longitudinal aging study. J Am Med Dir Assoc.

[28] Lim S, Kim JH, Yoon JW (2010). Sarcopenic obesity: prevalence and association with metabolic syndrome in the Korean longitudinal study on health and aging (KLoSHA). Diabetes Care.

[29] Gao L, Jiang J, Yang M, Hao Q, Luo L, Dong B (2015). Prevalence of sarcopenia and associated factors in Chinese community-dwelling elderly: Comparison between rural and urban areas. J Am Med Dir Assoc.

[30] Volpato S, Bianchi L, Cherubini A (2014). Prevalence and clinical correlates of sarcopenia in community-dwelling older people: application of the EWGSOP definition and diagnostic algorithm. J Gerontol A Biol Sci Med Sci.

[31] Mitchell WK, Williams J, Atherton P, Larvin M, Lund J, Narici M (2012). Sarcopenia, Dynapenia, and the Impact of Advancing Age on Human Skeletal Muscle Size and Strength; a Quantitative Review. Frontiers in Physiology.

[32] Albani D, Batelli S, Polito L (2009). A polymorphic variant of the insulin-like growth factor 1 (IGF-1) receptor correlates with male longevity in the Italian population: A genetic study and evaluation of circulating IGF-1 from the “Treviso Longeva (TRELONG)” study. BMC Geriatr.

[33] Looker A, Flegal K, Melton LR (2007). III impact of increased overweight on the projected prevalence of osteoporosis in older women. Osteoporos Int.

[34] Sioen I, Lust E, De Henauw S, Moreno LA, Jiménez-Pavón D (2016). Associations between body composition and bone health in children and adolescents: a systematic review. Calcif Tissue Int.

[35] Srikanthan P, Hevener AL, Karlamangla AS (2010). Sarcopenia exacerbates obesity-associated insulin resistance and dysglycemia: findings from the National Health and Nutrition Examination SurveyIII. PLoSOne.

[36] Leenders M, Verdijk LB, van der Hoeven L (2013). Patients with type 2 diabetes patients show a greater decline in muscle mass, muscle strength, and functional capacity with aging. J Am Med Dir Assoc.

[37] Kalyani RR, Saudek CD, Brancati FL, Selvin E (2010). Association of diabetes, comorbidities, and A1c with functional disability in older adults: results from the national health and nutrition examination survey (NHANES),1999-2006. Diabetes Care.

[38] Lim HS, Park YH, Suh K (2018). Association between sarcopenia, Sarcopenic obesity, and chronic disease in Korean elderly. J Bone Metab.

[39] Scott D, Cumming R, Naganathan V (2018). Associations of sarcopenic obesity with the metabolic syndrome and insulin resistance over five years in older men: the Concord health and ageing in men project. Exp Gerontol.

[40] Cleasby ME, Jamieson PM, Atherton PJ (2016). Insulin resistance and sarcopenia: mechanistic links between common co-morbidities. J Endocrinol.

[41] Schrager MA, Metter EJ, Simonsick E (1985). Sarcopenic obesity and inflammation in the InCHIANTI study. J Appl Physiol.

[42] Baumgartner RN, Waters DL.: Sarcopenia and sarcopenic-obesity. In: Pathy, MSJ. (eds.) Principles and practice of geriatric medicine, pp. 909–933. London (2005).

[43] Rolland YM, Perry HM, Patrick P, Banks WA, Morley JE (2007). Loss of appendicular muscle mass and loss of muscle strength in young postmenopausal women. J Gerontol A Biol Sci Med Sci.

[44] Picetti D, Foster S, Pangle AK (2017). Hydration health literacy in the elderly. Nutr Healthy Aging.

